# From single-cell to spatial transcriptomics: decoding the glioma stem cell niche and its clinical implications

**DOI:** 10.3389/fimmu.2024.1475235

**Published:** 2024-09-17

**Authors:** Lei Cao, Xu Lu, Xia Wang, Hao Wu, Xiaye Miao

**Affiliations:** ^1^ Department of Oncology, The Affiliated Suqian First People’s Hospital of Nanjing Medical University, Suqian, China; ^2^ Department of Oncology, The Affiliated Huai’an Hospital of Xuzhou Medical University and the Second People’s Hospital of Huai’an, Huai'an, China; ^3^ Department of Laboratory Medicine, Northern Jiang su People's Hospital, Yangzhou, Jiangsu, China

**Keywords:** cancer stem cells, spatial transcriptomics, single-cell RNA sequencing, prognostic signature, TUBA1C

## Abstract

**Background:**

Gliomas are aggressive brain tumors associated with a poor prognosis. Cancer stem cells (CSCs) play a significant role in tumor recurrence and resistance to therapy. This study aimed to identify and characterize glioma stem cells (GSCs), analyze their interactions with various cell types, and develop a prognostic signature.

**Methods:**

Single-cell RNA sequencing data from 44 primary glioma samples were analyzed to identify GSC populations. Spatial transcriptomics and gene regulatory network analyses were performed to investigate GSC localization and transcription factor activity. CellChat analysis was conducted to infer cell-cell communication patterns. A GSC signature (GSCS) was developed using machine learning algorithms applied to bulk RNA sequencing data from multiple cohorts. *In vitro* and *in vivo* experiments were conducted to validate the role of TUBA1C, a key gene within the signature.

**Results:**

A distinct GSC population was identified, characterized by high proliferative potential and an enrichment of E2F1, E2F2, E2F7, and BRCA1 regulons. GSCs exhibited spatial proximity to myeloid-derived suppressor cells (MDSCs). CellChat analysis revealed an active MIF signaling pathway between GSCs and MDSCs. A 26-gene GSCS demonstrated superior performance compared to existing prognostic models. Knockdown of TUBA1C significantly inhibited glioma cell migration, and invasion *in vitro*, and reduced tumor growth *in vivo*.

**Conclusion:**

This study offers a comprehensive characterization of GSCs and their interactions with MDSCs, while presenting a robust GSCS. The findings offer new insights into glioma biology and identify potential therapeutic targets, particularly TUBA1C, aimed at improving patient outcomes.

## Introduction

Gliomas are the most common and aggressive primary tumors affecting the central nervous system. According to data from the Central Brain Tumor Registry of the United States (CBTRUS) for the years 2013 to 2017, these neoplasms accounted for approximately 25% of all adult primary brain tumors and 81% of malignant central nervous system tumors in the United States (1). The National Comprehensive Cancer Network (NCCN) Guidelines classify glioma as a diverse group of neoplasms. Ranging from low-grade gliomas (LGGs), such as surgically treatable pilocytic astrocytomas to highly invasive and virtually incurable glioblastoma multiforme (GBM) (2). Despite extensive research into molecular therapies targeting oncogenic pathways and immune checkpoints in gliomas, significant improvements in patient outcomes have remained elusive. This situation underscores the necessity for continued investigation into novel therapeutic approaches for this challenging group of tumors (3, 4).

Glioma stem cells (GSCs), characterized by their stem cell attributes, constitute a minor subset within the larger glioma cell population. The majority of the glioma mass comprises differentiated progeny, a characteristic conferred upon GSCs due to the capability for self-renewal (5). Within the cancer stem cells (CSCs) milieu, immune cells such as cancer-associated fibroblasts (CAFs), tumor-associated macrophages (TAMs), and myeloid-derived suppressor cells (MDSCs) secrete cytokines like TGFβ significantly contributing toward the EMT-mediated invasion of CSCs (5, 6). Contemporary research indicates a significant role of CSCs in glioma recurrence and resistance to chemoradiotherapy (6, 7). Targeting CSCs and their supportive microenvironment has emerged as a promising strategy for developing novel and more effective treatments for gliomas, with the aim of improving patient outcomes and addressing the challenge of recurrence. Flow cytometry or magnetic techniques facilitate the enrichment of CSC populations from bulk tumors, leveraging specific cell surface markers for selection. However, the expression of specific markers is not consistently observed across all glioma stem cells. It is essential to recognize that CSCs, when exposure to novel microenvironments, are susceptible to alterations in their state, a phenomenon observed regardless of the methodology used for CSC enrichment. Notably, *in vitro* culture conditions can prompt variations in surface marker expression and modulate the intrinsic biological states of glioma cells (8). Consequently, examining CSCs without prior selection provides a valuable opportunity to investigate their inherent characteristics, potentially yielding insights into the processes by which CSCs originate and differentiate into the cells implicated in gliomagenesis.

The enhanced resolution for cell-type identification and characterization, previously unattainable with bulk RNA sequencing (RNA-seq), has been facilitated by Single-cell RNA sequencing (scRNA-seq) (9, 10). Numerous researchers have already leveraged the advantages of single-cell sequencing to successfully discover cancer biomarkers and identify potential therapeutic targets (10–14). A primary objective of scRNA-Seq research is to decipher the hierarchical differentiation structures of complex tissues (15). To achieve this, it necessitates an unbiased measure of the differentiation potential of individual cells, thus enabling the recognition of either stem or multipotent progenitor cells and the establishment of a ranking of individual cells along potency gradients of differentiation (16). However, current in silico methods present challenges in differentiating between adult stem cells with long-term regenerative capabilities and more differentiated cells. Although models based on gene expression possess the potential to surmount these constraints (17), the extent of their applicability across varied developmental systems and an array of single-cell sequencing methodologies remains to be fully elucidated.

In this investigation, we developed a novel framework consisting of five distinct algorithms specifically designed to identify GSCs using scRNA-seq data. Furthermore, we utilized the SCENIC algorithm and spatial transcriptomics (ST) analysis to elucidate the transcription factor (TF) activities and cell communications within these GSCs. Bulk RNA-seq deconvolution highlighted the significant role of GSCs in predicting poor patient prognosis. Based on a comprehensive combination of 429 algorithmic combinations, we established a GSC Signature (GSCS). Ultimately, we conducted *in vivo* and *in vitro* experiments to empirically validate the malignant characteristics associated with TUBA1C, a gene that identified as the most critical component within the GSCS.

## Methods

### Collection and pre-processing of scRNA-seq data

ScRNA-seq data from 44 primary glioma samples was obtained from a previous study (18). To ensure data quality, single cells expressing fewer than 500 expressed genes, over 20% mitochondrial transcripts, or containing more than 50% ribosomal transcripts were excluded from further analysis. Additionally, we removed genes that were expressed in fewer than three single cells. We used the DoubletFinder Python package was utilized to identify potential doublets (19). The filtered dataset consisted of 22,8156 cells, which were analyzed using (20). We normalized gene expression using Seurat’s LogNormalize method, applying a scale factor of 10,000 (21). Highly variable genes (n=2000) were selected and their expression values were scaled prior to principal component analysis (PCA). Batch effects were corrected using the Harmony R package (22). Data analysis was performed using functions from the Harmony and Seurat R packages, including NormalizeData, FindVariableFeatures, ScaleData, RunPCA, FindNeighbors, FindClusters, and RunUMAP. Cell cycle phases were scored using Seurat’s CellCycleScoring function (23).

### InferCNV analysis

To assess the score of large-scale chromosomal copy number variations (CNVs) in somatic cells, the inferCNV R package (24) was utilized. A raw counts matrix, an annotation file, and a gene/chromosome position file were prepared according to the specified data prerequisites (https://github.com/broadinstitute/inferCNV). T/NK cells and B cells were subsequently designated as the reference cells for the analysis.

### Identification of GSCs

We developed a novel framework that encompasses five distinct algorithms—CCAT (16), CytoTRACE (17), Monocle3 (25), PAGA (26), and Slingshot (27)—each meticulously designed to assess differentiation capacity of cells using sscRNA-seq data. CCAT, based on the concept of entropy-rate, was executed through the SCENT R package (16). In contrast, CytoTRACE provides an unsupervised framework for predicting relative differentiation states from single-cell transcriptomes, utilizing the CytoTRACE R package (17). An increase in scores calculated by both CCAT and CytoTRACE indicates a higher degree of cellular differentiation. Pseudotime analysis was conducted using the Monocle3 R package, the Slingshot R package, and the PAGA method in the SCANPY Python package (27). The framework code can be accessed at the following GitHub repository: https://github.com/Caolab2024/Cancer_stem_cells/tree/main.

### Abundance of cell types in bulk RNA-seq data

Cell type abundances were estimated from the bulk kidney expression data in the TCGA cohort using the BisqueRNA R package (28). A PCA-based method was employed to deconvolute the seven primary kidney cell types based on scRNA data for subsequent analyses.

### Gene regulatory network analysis

We applied SCENIC (29), a novel computational approach for inferring regulatory networks and identifying TFs from scRNA-seq data, to individual cells. Subsequently, we employed receiver operating characteristic curve (ROC) analysis to identify regulons that were preferentially expressed in distinct cell clusters based on transcription factors or their target genes.

### Processing of glioma spatial transcriptome sequencing data and inferring cellular localization

We obtained spatial transcriptome data from 28 specimens available in the OSF repository(https://osf.io/4q32e/), comprising a total of 88,793 spots. Using the Seurat, an R package for single-cell genomics (20), we performed the following steps: (1) normalized and scaled the UMI counts using SCTransform and identified the most variable features; (2) reduced dimensionality and clustered the spots with RunPCA, applying the default parameters and the top 30 principal components.

To deconvolve the transcriptome data into cell-type-specific gene expression profiles, we utilized RCTD (30), a computational method for resolving cell types in complex biological samples. RCTD can help uncover the cellular composition, function and interactions in biological research.

We estimated cell-type dependencies using MISTy (31), a method for inferring mutual information between cell types. MISTy was applied to the RCTD estimates from all slides, and utilizing a multi-view model with a parameter that weighted the estimates of neighboring cell types (effective radius = 15 spots). We interpreted the median standardized importances of each view across all slides as indicators of cell-type colocalization or mutual exclusion in various spatial contexts.

CellChat analysis was performed using the CellChat R package (32). This tool enables the inference and analysis of cell-cell communication networks from single-cell RNA sequencing data. By utilizing CellChat, we were able to explore intercellular signaling pathways and identify key communication patterns among distinct cell populations.

### Collection and pre-processing of bulk RNA-seq data

We searched several public databases, including The Cancer Genome Atlas (TCGA, http://portal.gdc.cancer.gov/),ArrayExpress(https://www.ebi.ac.uk/biostudies/arrayexpress), Chinese Glioma Genome Atlas (CGGA, http://www.cgga.org.cn/), and Gene Expression Omnibus (GEO, https://www.ncbi.nlm.nih.gov/geo/) for datasets that met the following criteria: (1) more than 60 samples per cohort; (2) Affymetrix Human Genome U133 Plus 2.0 Array or high-throughput sequencing platforms; and (3) primary tumors from patients who did not receive any treatments before resection. We obtained a total of 2285 samples from 6 cohorts: TCGA-GBMLGG (n = 683) (33), CCGA1 (n = 413) (34), CCGA2 (n = 273) (34), GSE16011 (n = 262) (35), GSE108474 (n = 490) (36), and E-MTAB-3892 (n =164) (37). Transcripts per million (TPM) data for TCGA-GBMLGG were downloaded from UCSC Xena database. For data generated using the Affymetrix Human Genome U133 Plus 2.0 Array, we applied the robust multiarray averaging (RMA) algorithm from the Affy R package for preprocessing. Finally, We log2 transformed, z-score normalized, and removed batch effects from the gene expression data across all cohorts using the surrogate variable analysis (SVA) algorithm (38).

### Development of signatures using an artificial intelligence network

We sought to develop a precise and robust GSCS for predicting glioma patient prognosis. To achieve this, we built an artificial intelligence network incorporating 429 algorithm combinations, integrating 27 algorithms from traditional regression, machine learning, and deep learning approaches. These algorithms included CoxTime, DeepSurv, DeepHit, Logistic-Hazard, PC-hazard, Akritas, Coxboost, VSOLassoBag, RSF, GBM, SuperPC, obliqueRSF, CForest, GLMBoost, BlackBoost, Rpart, Survreg, Ranger, Ctree, LASSO, plsRcox, survival-SVM, Ridge, Enet, XGBoost, Boruta, and stepwise Cox. Initially, we conducted univariate Cox regression to identify prognostic GSC markers in the TCGA cohort at a significance level of *P* < 0.05. Subsequently, we applied the 429 algorithm combinations to these markers to develop predictive models within the TCGA cohort. The predictive performance of each algorithm combination was evaluated using C-indices across all validation cohorts. The optimal algorithm combination was selected based on the highest mean C-index. We stratified glioma patients into high- and low-risk groups according to the optimal cutoff value obtained by the survminer R package. The network code can be accessed at the following GitHub repository: https://github.com/Caolab2024/Cancer_stem_cells/tree/main.

### Functional annotation of the GSCS

We conducted gene set variation analysis (GSVA) and gene set enrichment analysis (GSEA) using the MSigDB database employing the GSVA and clusterprofiler (39, 40) R packages. The differentially expressed genes (log2FC > 1, adjusted *P <*0.05) between the low- and high-risk groups were subjected to Metascape for enrichment analysis (41).

### Cell transfection and RT-qPCR

Cell transfection was conducted using two distinct siRNAs targeting TUBA1C, synthesized by Ribobio (Guangzhou, China) and delivered with Lipofectamine 3000 (Invitrogen, USA). The siRNA sequences are provided in **Supplementary Table 1**. RNA extraction from tissues or cell lines was performed using TRIzol (Thermo) followed by cDNA synthesis with the PrimeScript™RT kit preceded gene expression quantification was then carried out via SYBR qPCR Master Mix on the Roche LightCycler 480 (Roche, GER) (42). Primer sequences were obtained from Tsingke Biotech (Beijing, China) and detailed in **Supplementary Table 1**.

### Cell counting

In each well of the 96-well plates, two thousand treated cells were seeded, followed by the addition of the CCK-8 labeling reagent for further processing (43). Observations and assessments were conducted on days 0, 1, 2, 3, 4, and 5 to monitor the cell responses and outcomes.

### Colony formation

Transfected LN299 and U87 cells (siNC, siTUBA1C-1, siTUBA1C-2 groups) were seeded at a density of 1,000 cells per well in a 6-well plate and incubated for 14 days to allow colony formation. The medium was replaced every 3 days. After incubation, the media were aspirated, and the cells were washed with phosphate-buffered saline (PBS). The cells were then fixed with 4% paraformaldehyde (PFA) for 20 minutes at room temperature, followed by staining with 0.5% crystal violet (Solarbio, China) for 20 minutes (44). After staining, excess crystal violet was rinsed off with water, and once dry, the number of colonies per well, defined as clusters of at least 50 cells, was counted. Colony numbers were compared across the siNC, siTUBA1C-1, and siTUBA1C-2 groups to evaluate the impact of gene silencing on colony formation.

### Wound healing

Following the transfection process, once cell confluence reached 95%, the transfected cells were seeded into 6-well plates. A sterile 200 μL pipette tip was used to draw a straight line, facilitating the gentle removal of unattached cells and debris with PBS. Subsequently, serum-free cell medium was replenished to sustain the cell culture. Photographs were captured at both the 0-hour and 48-hour time points at identical locations for comparative analysis.

### Transwell

Cells were seeded at a density of 2×10^4^ per well in 200 μL of serum-free medium within the upper chamber, which was either coated or left uncoated with matrix glue from BD Biosciences, USA. The lower chamber contained 700 μL of 10% complete medium. After a growth period of 36 hours, the cells were fixed, stained, and photographed for quantification.

### Subcutaneous tumor xenograft in nude mice

All mice were housed in the animal facility of Northern Jiangsu People’s Hospital Affiliated to Yangzhou University and maintained in a Specific-Pathogen Free (SPF) environment. The animal experiments were approved by the Ethics Committee of Northern Jiangsu People’s Hospital Affiliated to Yangzhou University. LN299 NC and Si-1 cell lines were cultured to the logarithmic growth phase, washed twice with PBS, and collected. The cell concentration was adjusted to 1×10^7^ cells/ml. A 100 μl cell suspension was subcutaneously injected into the right flank of each 6-week-old female nude mouse (BALB/c-nu), using 5 mice per group. Tumor length and width were measured every three days with calipers, and body weight was recorded. Tumor volume was calculated using the formula: Volume (mm³) = 0.5 × Length (mm) × Width (mm)². On day 21 post-injection, the mice were sacrificed, and the tumors were excised and weighed.

### Ki67 immunohistochemistry staining

The excised tumor tissues were fixed in 10% neutral formalin for 24 hours, followed by paraffin embedding and sectioning at a thickness of 4 μm. The sections were deparaffinized in xylene, rehydrated through a graded ethanol series, and endogenous peroxidase activity was blocked with 3% hydrogen peroxide. Antigen retrieval was performed using citrate buffer (pH 6.0) in a pressure cooker. After cooling to room temperature, the sections were blocked with goat serum for 30 minutes and then incubated overnight at 4°C with a Ki67 primary antibody (1:200 dilution, Abcam). The following day, the sections were washed three times with PBS for 5 minutes each, incubated with a secondary antibody for 30 minutes, developed with DAB, counterstained with hematoxylin, dehydrated, and mounted.

### Statistical analysis

Data analysis, statistics, and plotting were performed using R 4.3.1. Continuous variables were assessed using the Wilcoxon rank-sum test or the T test. The optimal cut-off value was determined with the survminer R package. Survival analysis was conducted using Cox regression and Kaplan-Meier methods via the survival R package. The pROC package was employed to implement the ROC curve for predicting binary categorical variables. The timeROC R package calculated the time-dependent area under the curve (AUC) for survival variables. Unless otherwise specified, *P* < 0.05 is considered statistically significant.

## Results

### Clustering and cell-type identification of single-cell RNA-seq data

**Figure 1:**
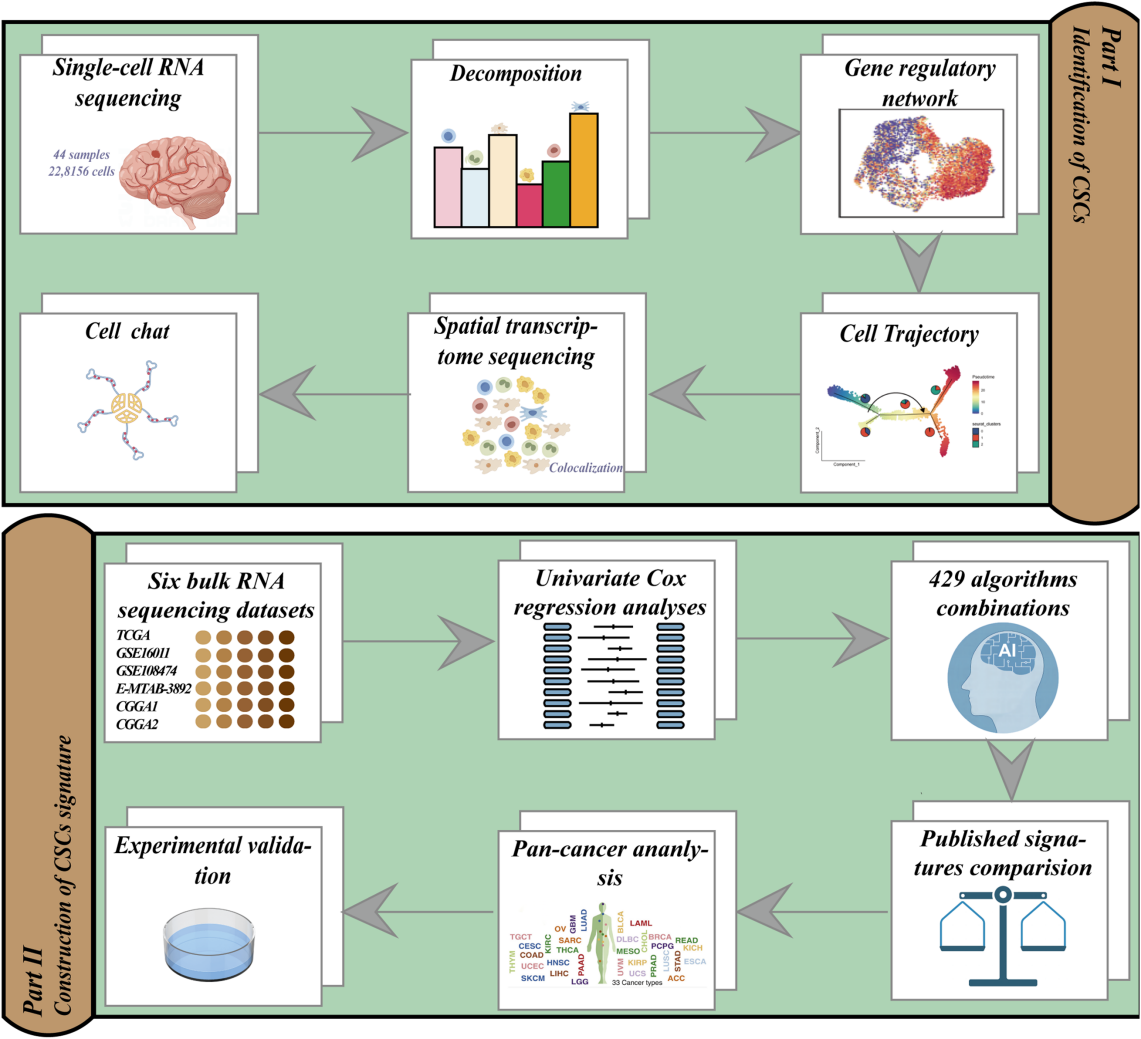
An illustration of the general workflow adopted in this study.

**Figure 2:**
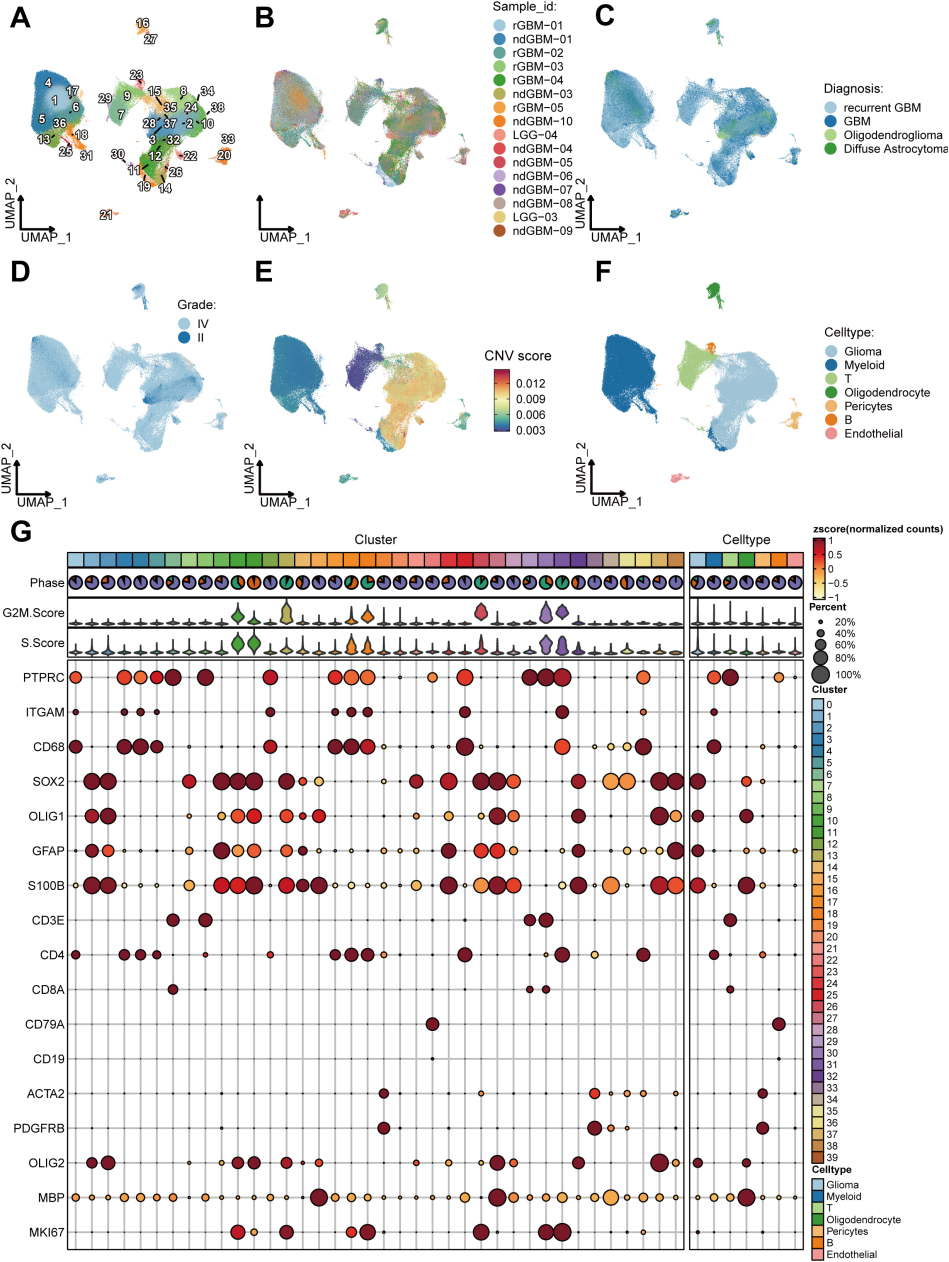
Clustering and cell-type identification of single-cell RNA-seq data. **(A–F)** UMAP projections of 228,156 aggregate single cells from 44 primary glioma samples showing the composition of different cell types in human gliomas. UMAP projections are shown by cluster numbers **(A)**, by the patient **(B)**, by pathological type **(C)**, by grade **(D)**, by Copy Number Variation (CNV) score **(E)** and by cell types **(F)**. **(G)** Dot plot showing marker gene expression and cell cycle score for different cell types.

The study flow diagram is shown in **Figure 1**. We analyzed 22,8156 cells from 44 samples that passed quality control steps to uncover the cellular and molecular heterogeneity of cancer cells in human gliomas. Unsupervised clustering identified 38 clusters with distinct gene expression patterns (**Figures 2A–D**). InferCNV analysis was employed to distinguish malignant and non-malignant cells based on the CNV score (**Figure 2E**). Each cluster was assigned to a cell type using InferCNV analysis and marker gene expression (**Figures 2F, G**).

### Identification and characterization of glioma stem cells

**Figure 3:**
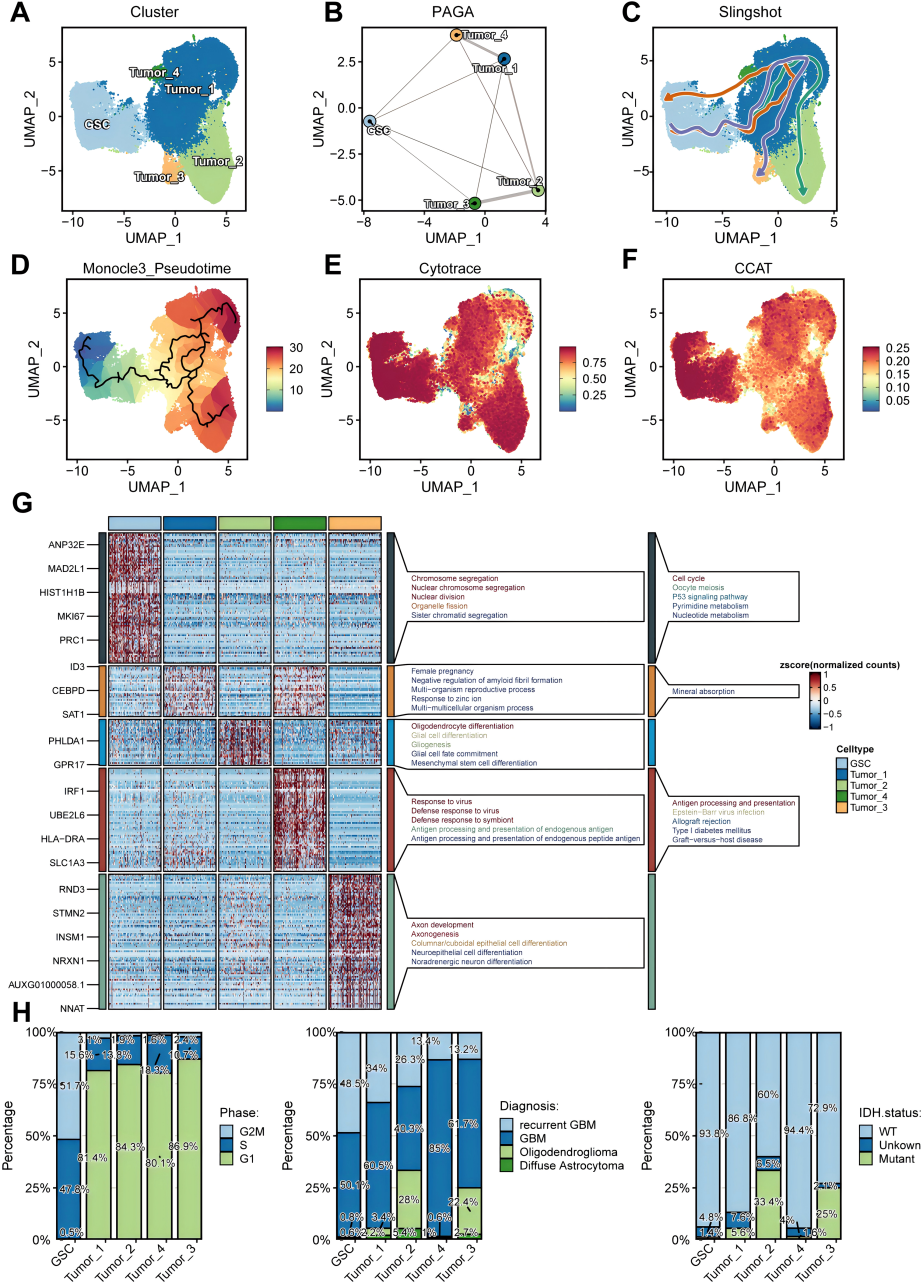
Identification and characterization of glioma stem cells (GSCs). **(A)** UMAP projections of 92,014 aggregate glioma cells are shown by cell annotation. **(B–D).** Trajectory inference using PAGA **(B)**, Slingshot **(C)**, and Monocle3 **(D)**. **(E, F)** UMAP projections of 92,014 aggregate glioma cells are shown by Cytotrace score **(E)** and CCAT score **(F)**. **(G)** Marker genes and Kyoto Encyclopedia of Genes and Genomes (KEGG)/Gene Ontology (GO) enrichment analysis for each glioma cell subpopulations. **(H)** Differential distribution of glioma cell subsets across cell cycle phases (left), histopathological classifications (middle), and IDH mutation status (right).

After conducting additional dimensionality reduction and clustering of glioma cells, our bespoke analytical framework enabled us to pinpoint GSCs, as indicated in **Figure 3A**. Utilizing the PAGA algorithm, we demonstrated the differential potential of GSCs as they evolve into various tumor cell subpopulations (**Figure 3B**). Analyses integrating Monocle3 and the Slingshot algorithm further elucidated the fundamental role that GSCs subpopulations play as the origin of tumoral evolution (**Figures 3C, D**). Both the CCAT and CytoTRACE algorithms highlighted the pronounced stem-like qualities inherent within GSCs (**Figures 3E, F**). Then, we performed a comprehensive enrichment analysis of the highly expressed marker genes across various tumor subpopulations, utilizing the Kyoto Encyclopedia of Genes and Genomes (KEGG) and Gene Ontology (GO) databases. Our findings revealed significant enrichment of GSC marker genes within pathways associated with cell proliferation, particularly those involving the cell cycle (**Figure 3G**). To further elucidate the proliferative capacity of GSCs, we conducted a detailed assessment of their cell cycle profiles, which demonstrated a predominant presence in the G2M and S phases (**Figure 3H**). This observation underscores the pivotal role of proliferation in the maintenance of stemness (45). Moreover, our analysis uncovered a striking prevalence of GSCs within samples of higher malignancy, including recurrent GBM, and gliomas with wild-type (WT) IDH (**Figure 3H**).

### GSCs correlated with unfavorable prognosis

**Figure 4:**
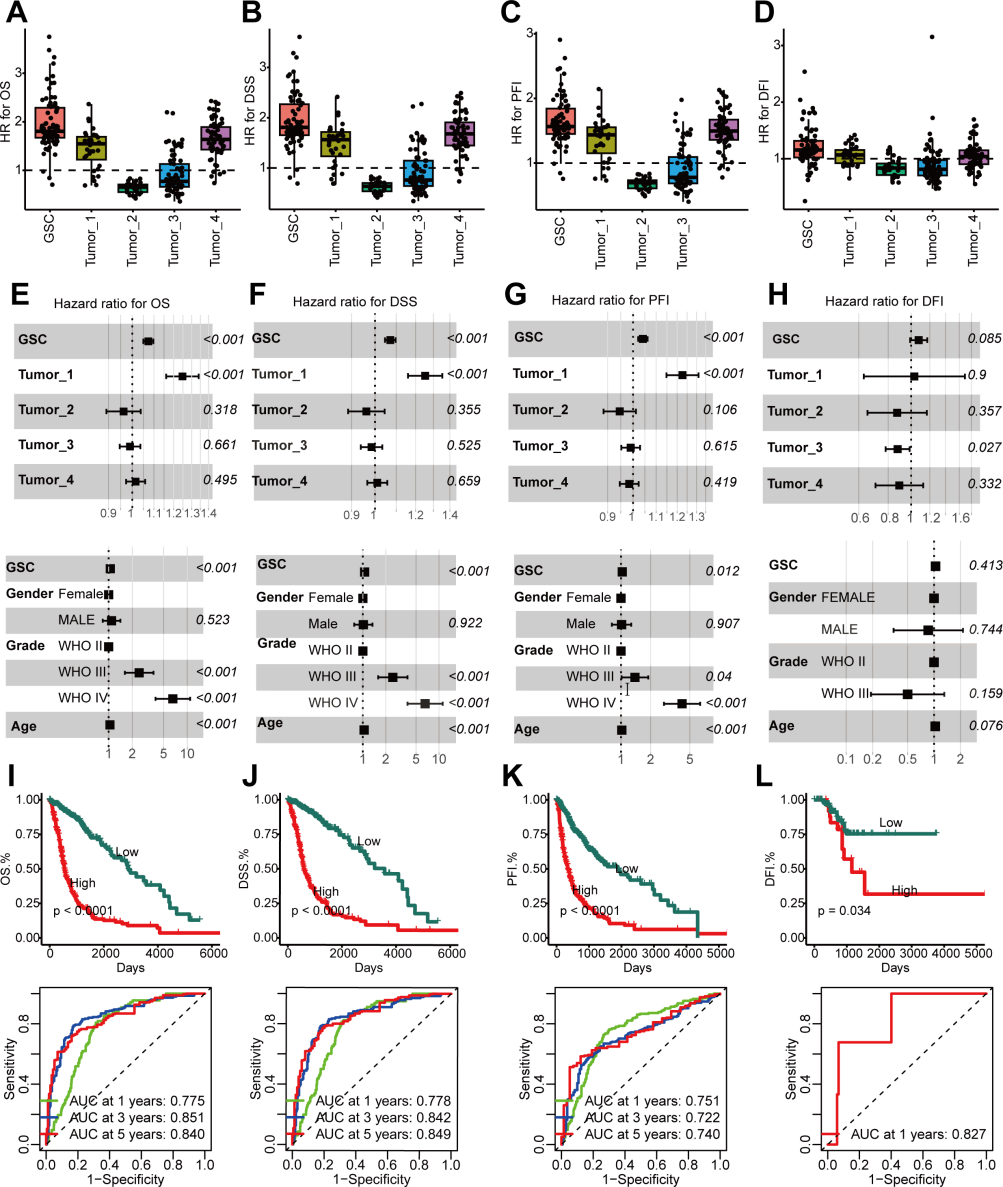
Higher GSCs abundance in glioma, linked with poor prognosis. **(A–D)** GSCs abundance’s association with poor overall survival (OS) **(A)**, disease-specific survival (DSS) **(B)**, progression-free interval (PFI) **(C)**, and disease free interval (DFI) **(D)** manifested through HR values for cell type marker genes from Cox proportional hazards regression. Each dot is a gene, with HR value on x-axis and cell type on y-axis. **(E–H).** Multivariate Cox analyses to identify the risk factors in OS **(E)**, DSS **(F)**, PFI **(G)**, DFI **(H)**. (**I–L)**. Time-dependent receiver operating characteristic (ROC) analysis (top) and Kaplan-Meier (KM) curves (below) of the GSCs abundance in OS **(I)**, DSS **(J)**, PFI **(K)**, DFI **(L)**.

Utilizing bulk RNA-seq data from the TCGA cohort, we evaluated the prognostic significance of five distinct tumor cell populations. Initially, we identified cell-type-specific marker genes at the single-cell level, characterized by a log2 fold change exceeding 1 and an adjusted *P* below 0.05, and proceeded to determine their hazard ratios (HRs) based on overall survival (OS), progression-free interval (PFI), disease-specific survival (DSS), and disease free interval (DFI) within the TCGA cohort. Our observations revealed that GSC marker genes exhibited the most elevated HRs for OS, PFI, DFI, and DSS (**Figures 4A–D**). We ascertained that these GSCs constitute independent prognostic indicators for OS, PFI, and DSS (*P* < 0.05) (**Figures 4E–G**). Nevertheless, GSCs did not emerge as independent risk factors for DFI, a finding potentially attributable to the paucity of DFI data (**Figure 4H**). Kaplan-Meier analysis further demonstrated that a higher abundance of GSCs was associated with adverse outcomes across OS, PFI, DFI, and DSS (*P* < 0.05) (**Figures 4I–L**). Moreover, the area under the curve (AUC) values for the prediction of OS, PFI, DSS, and DFI consistently surpassed 0.7 (**Figures 4I–L**), underscoring the formidable prognostic predictive power of GSCs.

### Unique TF profile associated with GSCs

**Figure 5:**
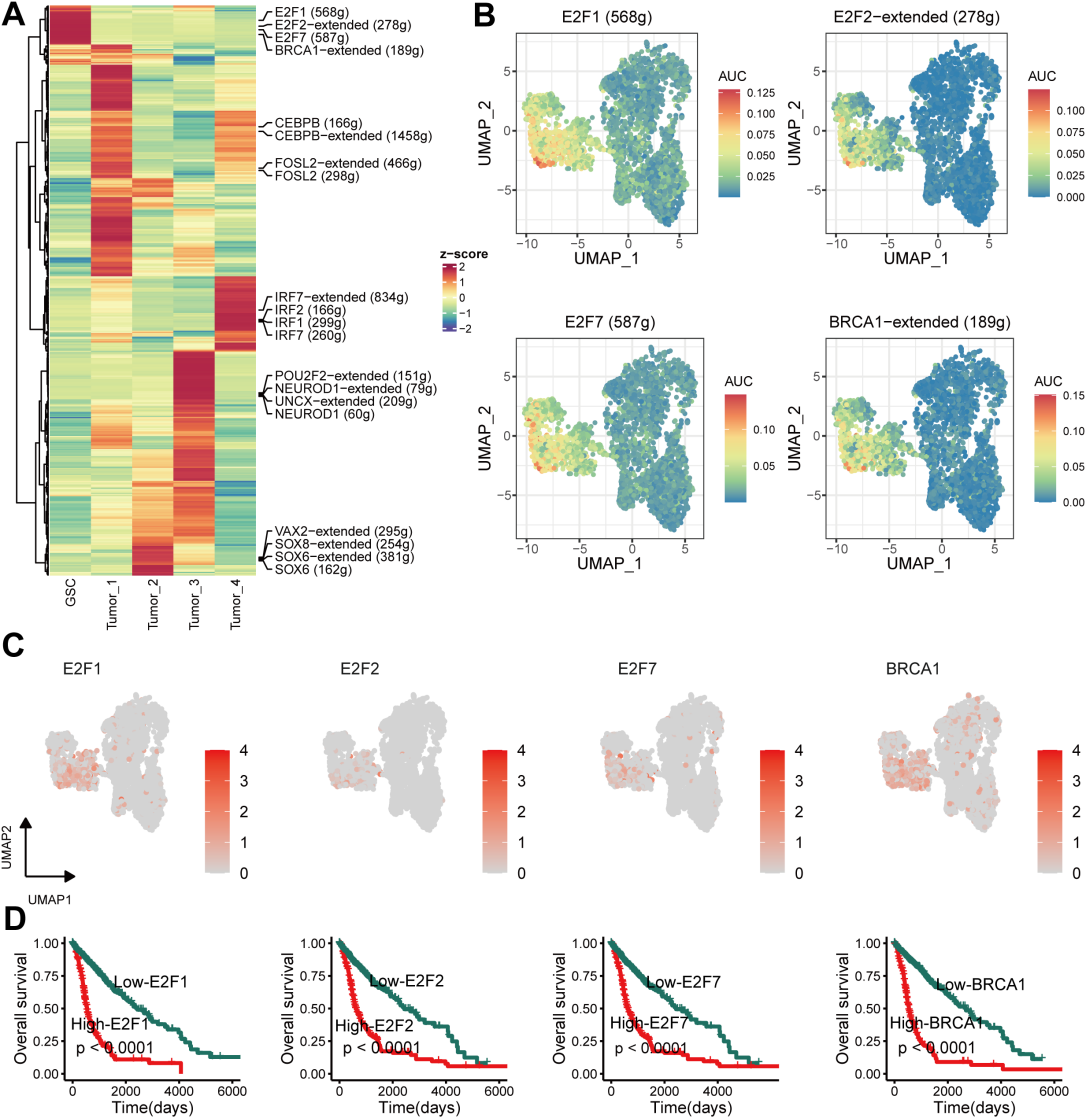
Unique transcription factor (TF) activity associated with GSCs. **(A)** Heatmap showing differences in TF activity scored by SCENIC. **(B)** TF activity of E2F1, E2F2, E2F7 and BRCA1 projected on UMAP. **(C)** TF expression of E2F1, E2F2, E2F7 and BRCA1 projected on UMAP. **(D)** Kaplan–Meier survival curves for E2F1, E2F2, E2F7 and BRCA1.

As previous studies (46) showed, TFs are key for cell fate specification. Each cell type exhibited a unique TFs pattern (**Figure 5A**). Strong enrichment was in E2F1, E2F2, E2F7, and BRCA1 regulon activity in GSCs (**Figure 5B**). **Figure 5C** displays the expression patterns of these four TFs, which correlated with worse prognosis in glioma patients from the TCGA cohort (*P* < 0.05) (**Figure 5D**). These findings indicated that the four TFs might be essential for maintaining the stemness of GSCs.

### Spatial transcriptomics of glioma reveals cellular localization

**Figure 6:**
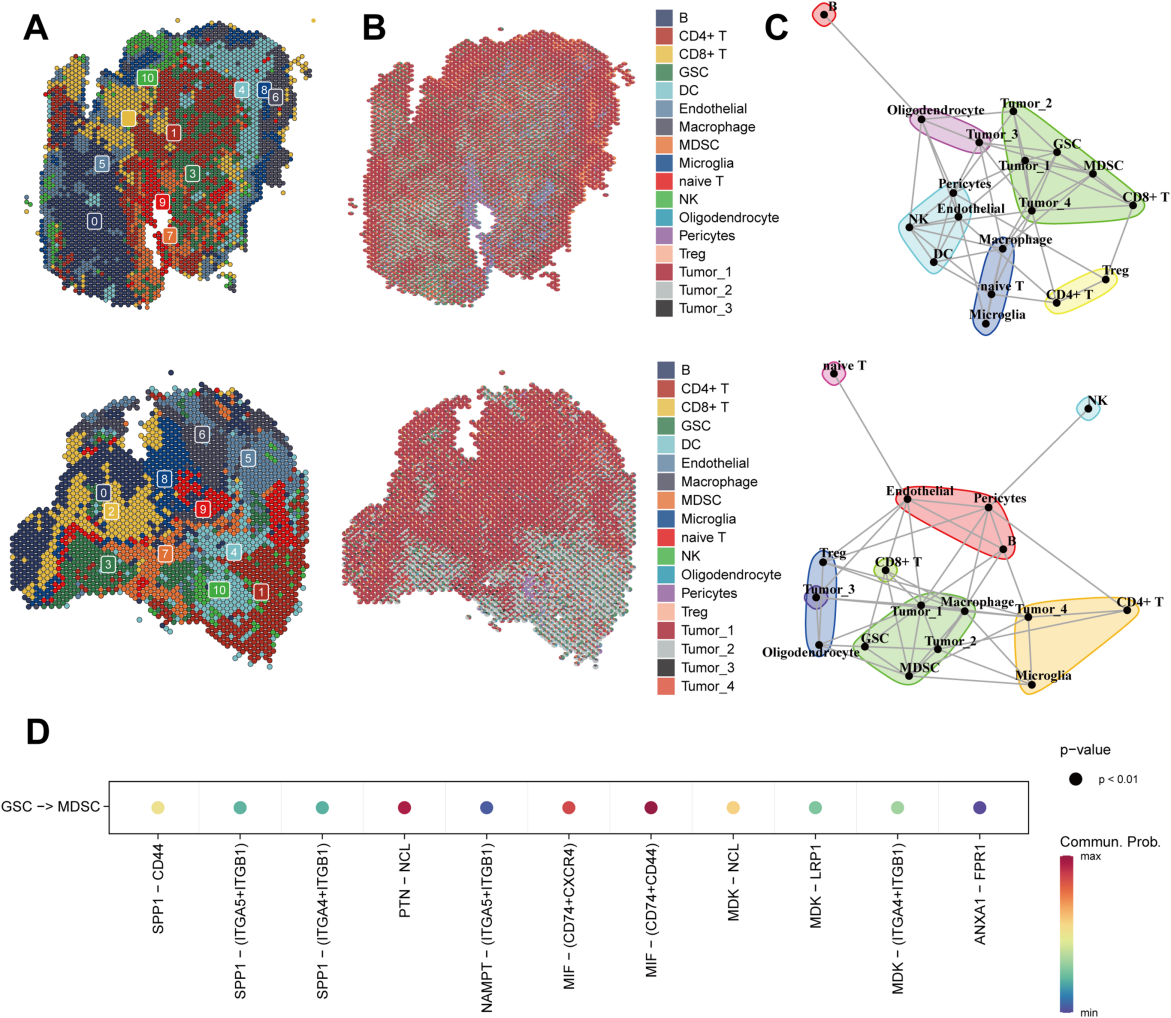
Spatial transcriptomics of glioma reveals cellular localization and cell communication. **(A)** Spatial Transcriptomics-based UMAP clustering of sample 1 (**top**) and sample 2 (**below**). **(B)** Spatial localization of individual clusters in the sample 1 (**top**) and sample 2 (**below**). **(C)** The spatial distribution of different cell-types in the spatial transcriptomics reference calculated by RCTD in the sample 1 (**top**) and sample 2 (**below**). **(D)** MIF signaling pathway from CellChat results.

We reduced the dimensionality and clustered the spatial transcriptome data, identified the predominant cell types at each spot based on the back-convolutional via the scRNA-seq reference data, and chose the two samples with the highest GSCs abundance for the next step in the analysis (**Figures 6A, B**). Based on MISTy results, GSCs had a close spatial location to myeloid-derived suppressor cells (MDSCs) (**Figure 6C**). Then, we found that the MIF pathway was active between GSCs and MDSCs (**Figure 6D**). This result confirmed the previous conclusion that GSCs activate MDSCs to suppress immune responses by secreting MIF (47).

### Construction of the GSCS

**Figure 7:**
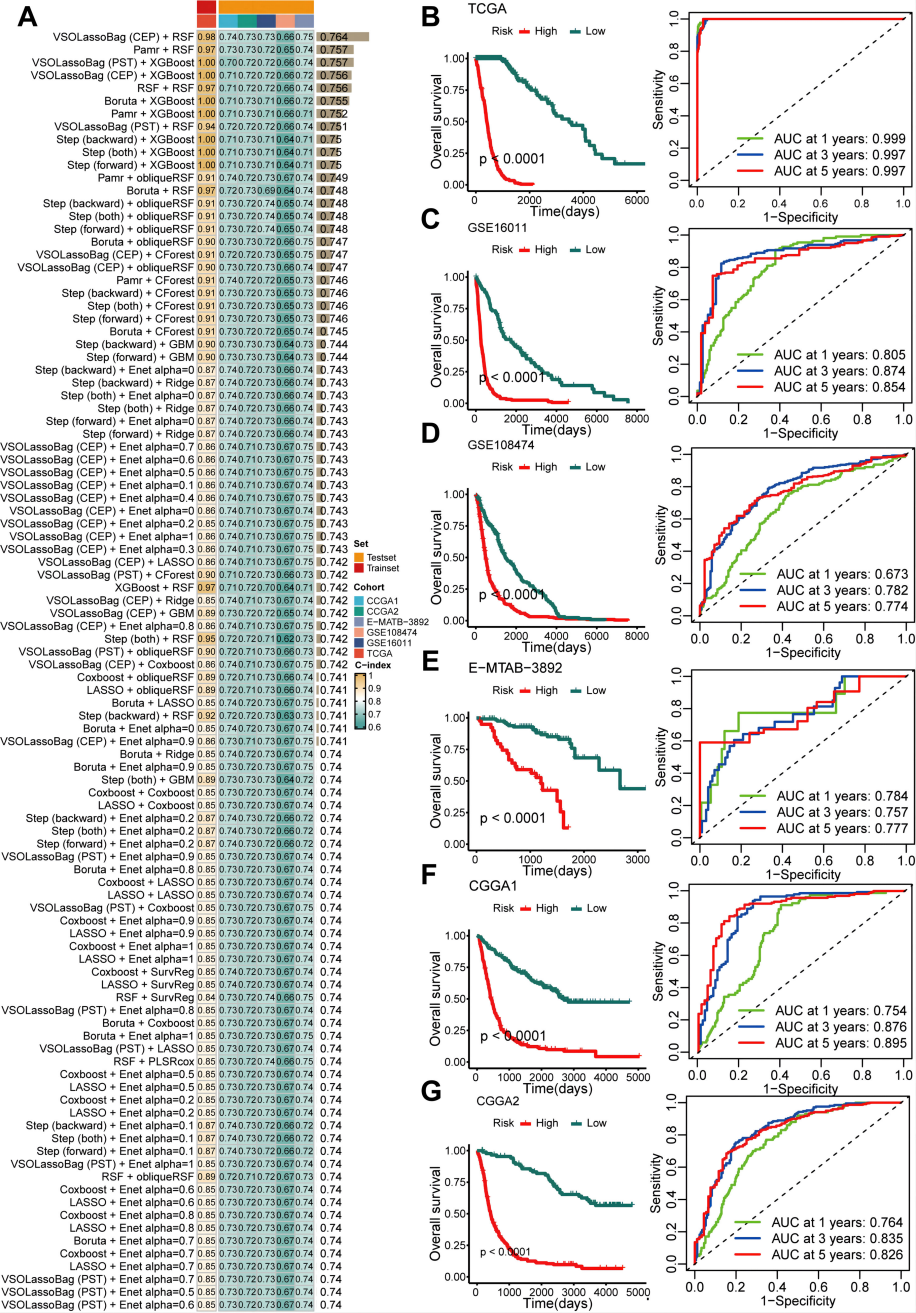
An artificial intelligence network was utilized to develop and validate a consensus GSC signature (GSCS). **(A)** A total of 429 prediction models were developed using a 10-fold cross-validation framework, and the C-index of each model was computed across all datasets. **(B–G).** Kaplan-Meier curves and ROC curves of OS according to the GSCS in the **(B)** TCGA, **(C)** GSE16011, **(D)** GSE108474, **(E)** E-MTAB-3892, **(F)** CGG1, and **(G)** CGGA2.

To further quantify the abundance of GSCs using key genes and improve the ability to predict prognosis in gliomas, we developed a GSCS on a novel artificial intelligence network. We first performed univariate Cox analysis to select the most specific GSCs marker (log2 fold change > 1, adjusted *P* = 0) with prognostic value (*P* < 0.05). We then fitted 429 algorithm combinations on the TCGA cohort and computed the C-index for each combination on the validation cohorts. The combination of VSOLassoBag and RSF had the highest mean C-index of 0.764 (**Figure 7A**). VSOLassoBag identified 26 genes, which were used by RSF to construct the GSCS (**Supplementary Figures 1A, B**). We stratified glioma patients into high- and low-risk groups based on the optimal cutoff from the TCGA cohort. Our results showed that the high-risk group had significantly worse OS than the low-risk group in all cohorts (*P* < 0.05) (**Figures 7B–G**). Moreover, time-dependent ROC curves demonstrated the robust and stable performance of the GSCS in the all cohorts (**Figures 7B–G**).

### Comparison of prognostic signatures

**Figure 8:**
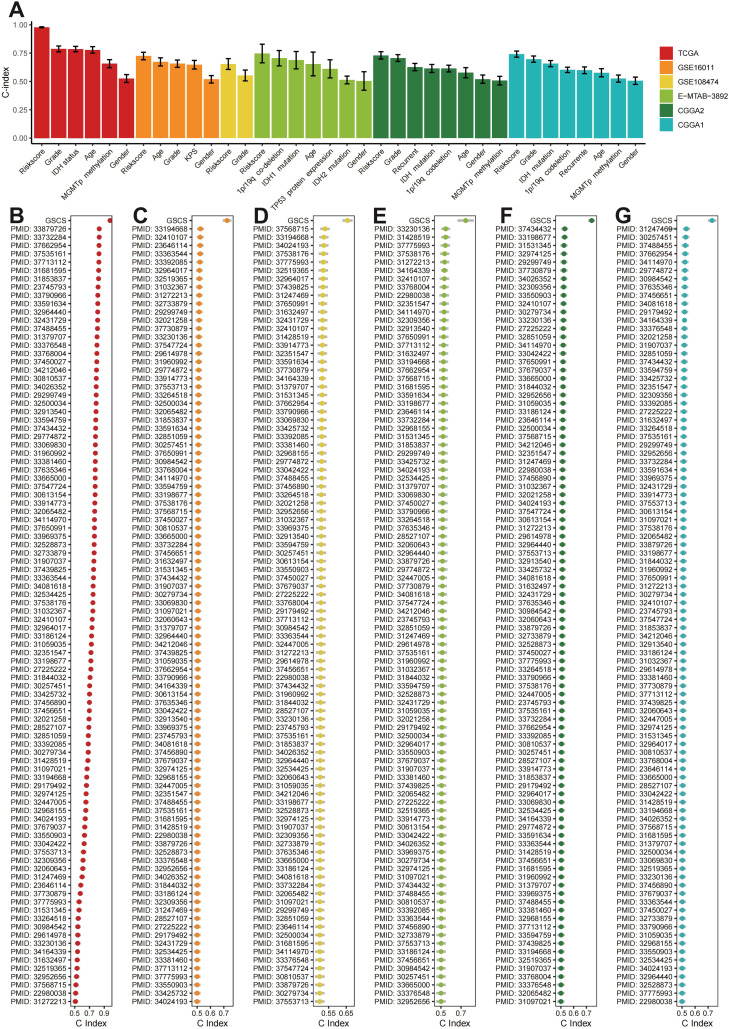
**(A)** The C-index of the GSCS and other models developed in the **(B)** TCGA, **(C)** GSE16011, **(D)** GSE108474, **(E)** E-MTAB-3892, **(F)** CGCA1, and **(G)** CGCA2.

The GSCS outperformed age, grade, gender, IDH status, MGMT promoter status, karnofsky score (KPS), 1p/19q co-deletion,TP53 protein expression, and recurrent status in terms of the C-index across the all cohorts (**Figure 8A**). We also compared the GSCS with other pulished signatures in the TCGA, GSE108474, GSE16011, E-MTAB-3892, CGCA1 and CGCA2 cohorts (**Figures 8B–H**). The GSCS had the highest C-index among all signatures in the all cohorts.

### The GSCS exhibited a generalizability signature in pan-cancer

**Figure 9:**
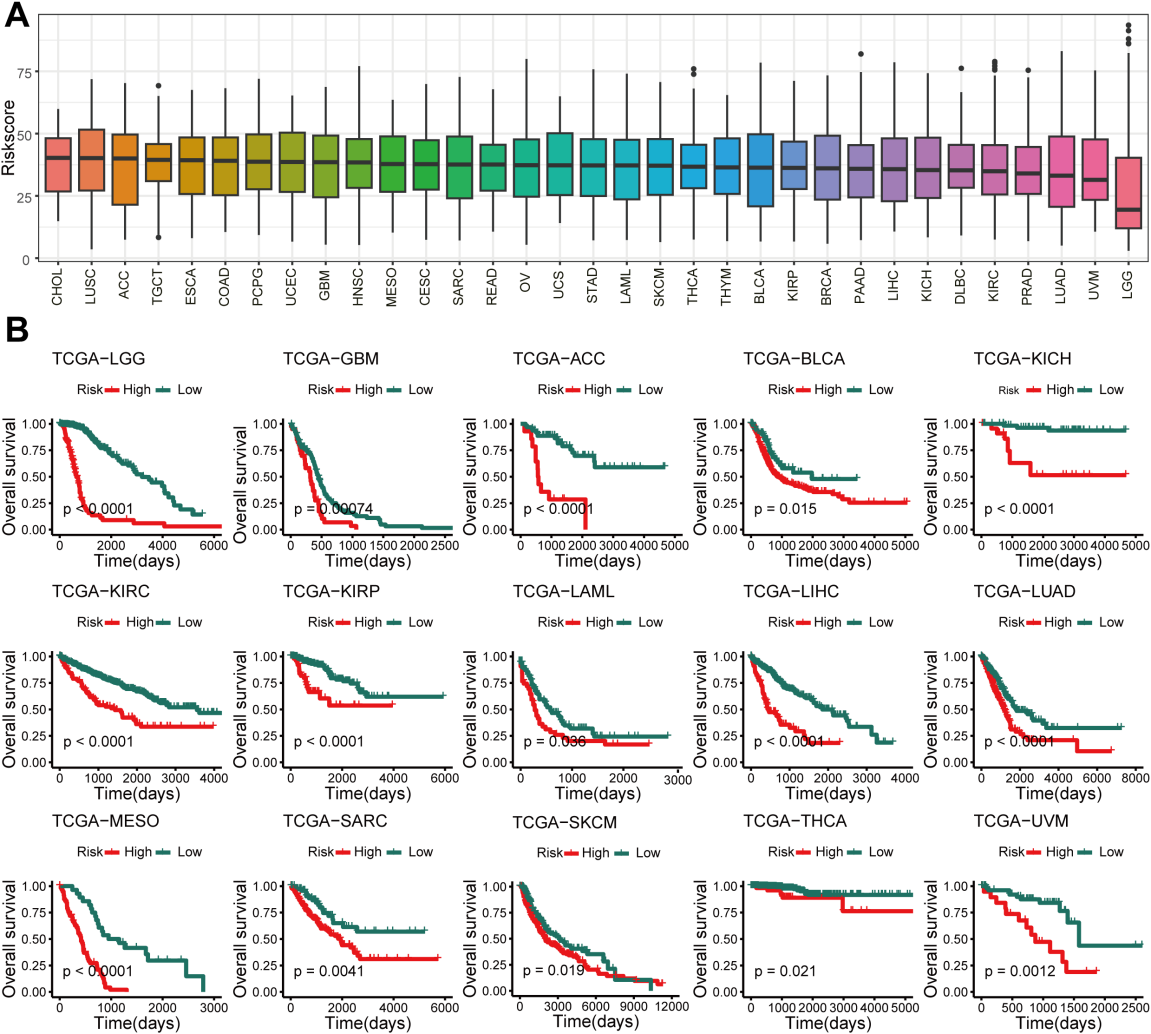
Pan-cancer of GSCS. **(A)** GSCS score for 33 cancers in the TCGA database. **(B)** Kaplan-Meier curves of OS according to the GSCS in the TCGA database.

We calculated the GSCS score for 33 cancers from the TCGA (**Figure 9A**) and divided them into High-risk and Low-risk groups. We observed that high GSCS scores correlated with worse prognosis across all 15 cancers (*P* < 0.05) (**Figure 9B**). These results suggest that GSCS may also be a key factor affecting the prognosis of various other cancers.

### Potential biological peculiarities of the GSCS

**Figure 10:**
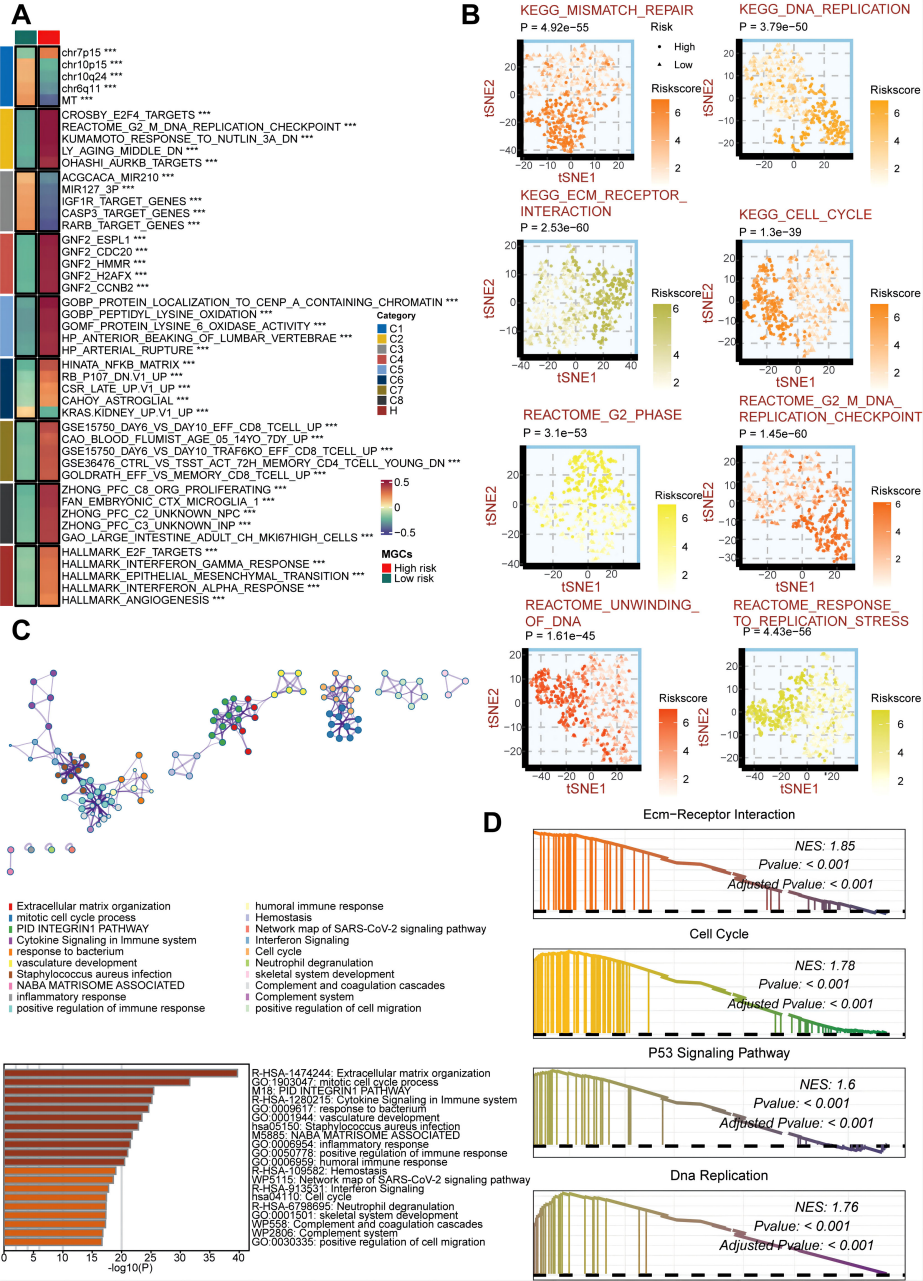
Biological peculiarities of the GSCS in the TCGA cohort. **(A)** MsigDB-based gene set variation analysis (GSVA) delineated the biological attributes of two risk groups. **(B)** t-Distributed Stochastic Neighbor Embedding (t-SNE) plots of kyoto encyclopedia of genes and genomes (KEGG) and reactome terms delineated the differences in pathway activity in the two risk groups. **(C)** Metascape-based enrichment analysis of differentially expressed genes between two risk groups. **(D)** Gene set enrichment analysis (GSEA) of KEGG terms for the GSCS. ***p < 0.001.

To explore the biological mechanisms underlying the association between GSCS and proliferative features, we performed pathway analysis on the GSCS score. We found that the score is strongly correlated with several tumorigenic pathways, such as the G2M DNA replication checkpoint, angiogenesis, E2F targets, and epithelial-mesenchymal transition (EMT) (*P* < 0.05) (**Figure 10A**). We also observed significant differences in proliferation-related pathways between the two risk groups (*P* < 0.05) (**Figure 10B**). The DEGs between the low- and high-risk groups were enriched in immune-related and proliferation-related pathways (*P* < 0.05) (**Figure 10C**). Furthermore, GSEA of kyoto encyclopedia of genes and genomes (KEGG) terms revealed that the high-risk group was enriched for ecm-receptor interaction, cell cycle, P53 signaling pathway, and DNA replication (*P* < 0.05) (**Figure 10D**). These findings also demonstrated the critical role of proliferation in maintaining stemness (48).

### The silence of the TUBA1C inhibits the malignant biological behavior of glioma cells

**Figure 11:**
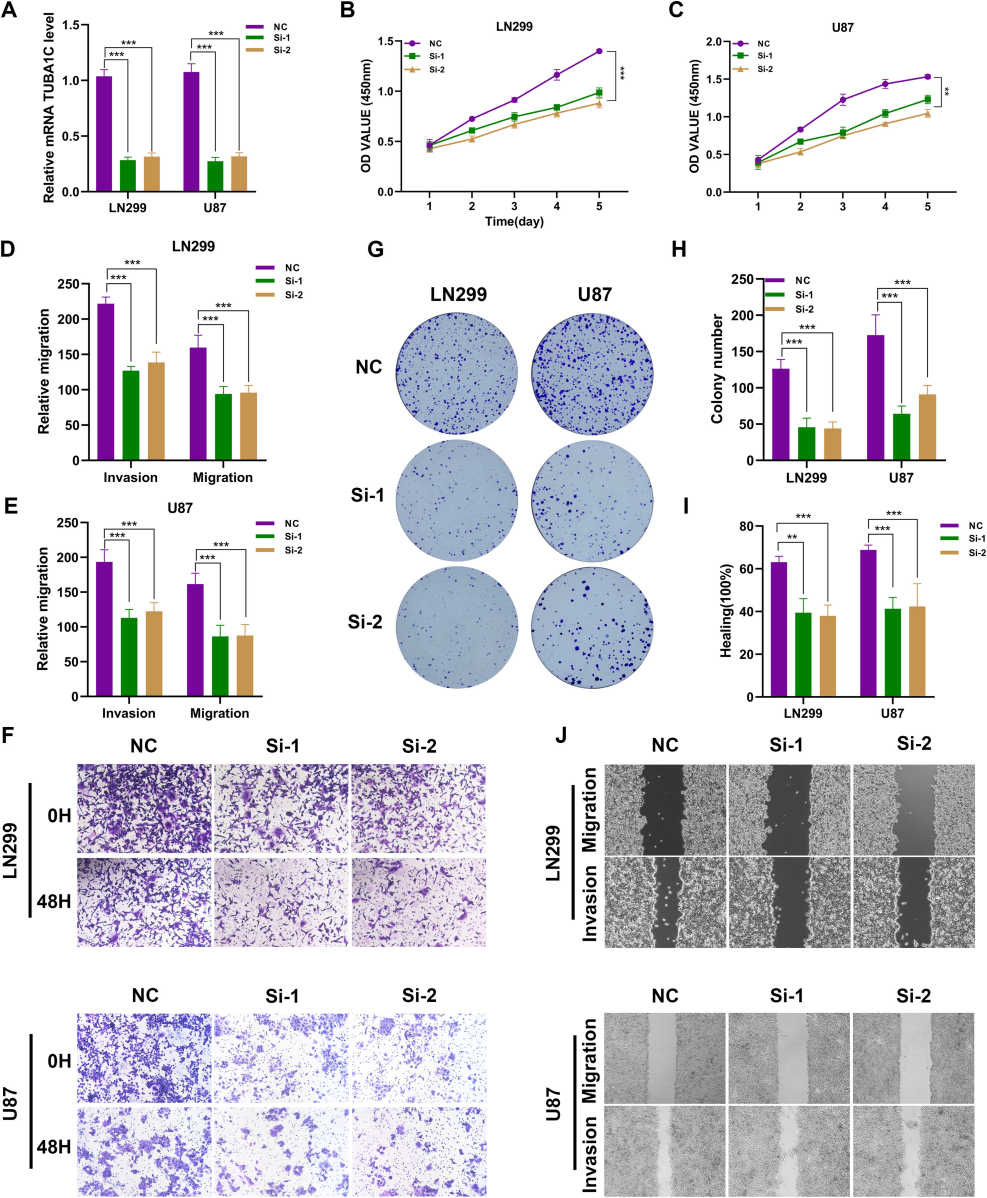
Silencing of TUBA1C Inhibits the Malignant Biological Behavior of Glioma Cells. **(A)** Schematic representation of the targeted siRNA-mediated knockdown of TUBA1C in LN299 and U87 glioma cell lines. **(B, C)** CCK-8 assay results showing a significant decrease in OD values of glioma cells following TUBA1C knockdown, indicating reduced cell viability. The results are presented as the mean ± SD of three independent experiments. **(D–F)** Transwell migration and invasion assays demonstrating a significant reduction in the migration and invasion abilities of glioma cells after TUBA1C knockdown. Quantitative analysis showed a significant decrease in the number of migrated and invaded cells. **(G, H)** Clonogenic assays confirming that the proliferation capacity of glioma cells is weakened upon TUBA1C knockdown. The number of colonies formed was significantly reduced compared to the control group. **(I, J).** Wound healing assays showing quantitative data that TUBA1C knockdown significantly slows the wound healing process, indicating a marked inhibition of cell migration capability. Wound closure percentage was significantly lower in TUBA1C knockdown cells. **p < 0.01; ***p < 0.001.

To investigate the key role of TUBA1C in the pathogenesis of glioma, we selected the LN299 and U87 glioma cell lines as research subjects and used targeted siRNA-mediated knockdown technology to regulate the expression of TUBA1C (**Figure 11A**). We evaluated the impact of TUBA1C knockdown on the viability of glioma cells using the CCK-8 assay. The experimental results showed that the OD value of glioma cells decreased significantly after TUBA1C expression was silenced (**Figures 11B, C**). To further investigate the impact of TUBA1C suppression on the migration and invasion ability of glioma cells, we conducted in-depth analyses. Using the Transwell migration and invasion assay, we observed that the invasion and migration ability of glioma cells were significantly reduced after TUBA1C knockdown (**Figures 11D–F**). Clonogenic assays confirmed that the proliferation ability of glioma cells was weakened after TUBA1C knockdown (**Figures 11G, H**). The wound healing experiment provided quantitative data, showing that TUBA1C knockdown significantly slowed the wound healing process, indicating that cell migration ability was significantly inhibited (**Figures 11I, J**). Taken together, we have revealed the key role of TUBA1C in promoting the proliferation, migration, and invasion of glioma cells. This discovery underscores the importance of TUBA1C as a potential therapeutic target in glioma treatment and may provide new ideas and methods for future glioma treatment.

### Silencing TUBA1C inhibits subcutaneous tumor growth *in vivo*

**Figure 12:**
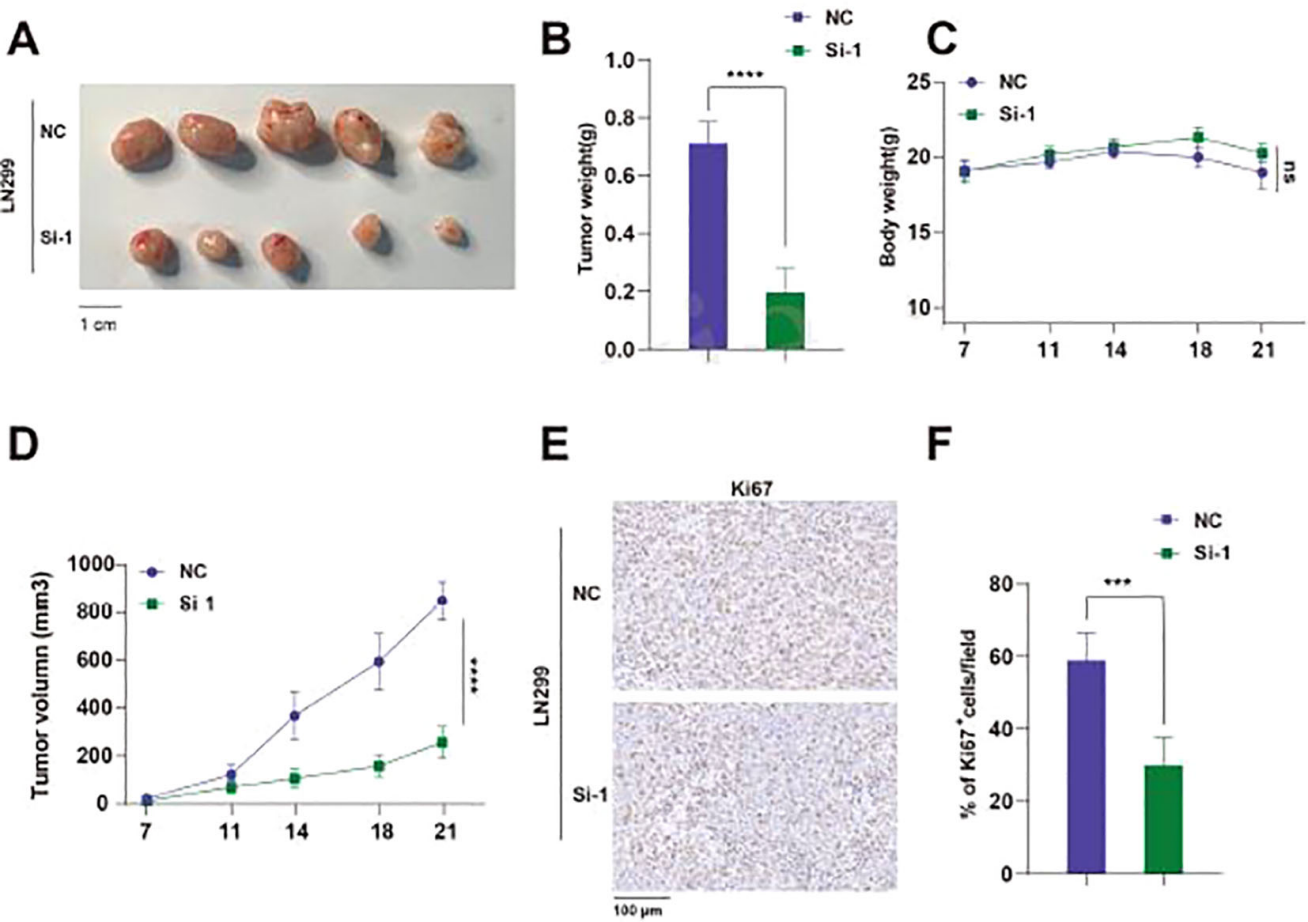
Knockdown of TUBA1C Inhibits Subcutaneous Tumor Growth *In Vivo*. **(A)** Representative images of tumors from mice, showing the tumor size comparison between the NC group and the Si-1 group. Scale bar: 1 cm. **(B)** Comparison of tumor weights between NC and Si-1 groups of mice. **(C)** Body weight changes during the experiment, showing no significant difference between the two groups. **(D)** Tumor volume growth curves, showing that the tumor volume in Si-1 group mice was significantly smaller than that in the NC group. **(E)** Ki67 immunohistochemistry images showing Ki67 positive cells in tumors from NC and Si-1 groups. **(F)** Comparison of the percentage of Ki67 positive cells per field. ****p < 0.0001.

We injected LN299 cell lines transfected with either TUBA1C-NC or TUBA1C-Si-1 subcutaneously into nude mice and dynamically observed the body weight and tumor growth of the mice. Through qPCR analysis, we compared the differences in TUBA1C expression between the two groups and confirmed the effectiveness of gene silencing in the xenograft tumors (**Supplementary Figure 2**). The results indicated that, compared to the NC group, the tumor weight and volume were significantly reduced in the Si-1 group mice, while there was no significant difference in body weight between the groups (**Figures 12A–D**). Immunohistochemical analysis of the mouse tissues showed that the percentage of Ki67 positive cells in the tumor tissues of the Si-1 group mice was also significantly reduced, suggesting that Si-1 treatment effectively inhibits tumor growth and cell proliferation (**Figures 12E, F**). In summary, these results highlight the potential of TUBA1C as a therapeutic target in glioma treatment strategies.

## Discussion

Treatment for gliomas has remained unchanged since 2005, involving surgical resection, radiation and concurrent and adjuvant TMZ (49). Adjuvant TMZ marginally increased survival time for adults with gliomas; however, this agent has caused systemic toxicities and decreased the quality of life for patients (49, 50). Moreover, many tumors exhibit primary or acquired resistance to temozolomide (51). The anti-angiogenic antibody bevacizumab, which neutralizes VEGF, was approved for recurrent gliomas and increased PFS in patients (52). However, subsequent trials showed no improvement in OS for newly diagnosed patients (53). While bevacizumab normalized the tumor blood vessels and reduced symptoms such as oedema, it does not extend lifespan (54). Gliomas are widely infiltrative and resistant to standard therapies, remaining non-curative. Therefore, new therapeutic options are urgently needed. In this work, based on integrated analysis of scRNA, bulk RNA sequencing, ST and machine learning algorithms, we identified and characterized GSC and developed a GSCS based on maker genes from the GSC as a predictive model for glioma patient outcomes.

We identified a glioma cell type, GSCs, that demonstrate a predominant presence in the G2M and S phases of the cell cycle. Furthermore, we observed a strong enrichment of E2F1, E2F2, E2F7, and BRCA1 regulons in GSCs. These four TFs are implicated in proliferation and DNA repair, indicating the high proliferative potential of this subcluster. Previous studies have shown that these TFs regulate stemness (55, 56). Additionally, GSCs were enriched with genes previously linked to poor glioma outcomes, such as CENPF (57), TOP2A (58), NUSAP1 (59), PTTG1 (60), UBE2C (61), and UBE2S (62). Unlike previous studies that focused on individual glioma genes, we employed a single-cell decomposition approach to reveal the association of the GSC type proportion and reduced survival. Moreover, the GSCs encompass not only encompasses known glioma genes, but also novel targets, such as HMGN2, TUBB4B, and ARL6IP1, that warrant further investigation. In summary, our research confirms the role of previously known genes in glioma GSCs while also identifying novel targets and TF regulators that can enhance our understanding of this complex disease. With further investigation, these discoveries could pave the way for developing more effective therapeutic strategies for glioma patients.

We aimed to enhance our understanding of the factors affecting survival in glioma patients, given their poor prognosis. Our findings suggest that GSCs could serve as useful biomarker for guiding treatment and predicting outcomes. Current prognostic tools for glioma primarily rely on factors such as WHO grade, IDH mutations, 1p/19q codeletion, MGMT promoter methylation, TERT promoter mutations and EGFR amplification (63). Cell type markers can aid in comprehend cancer biology and supplement existing clinical practices. The marker genes associated with GSCs may pave the way for new expression-based prognostic technologies. As RNA sequencing technology has matured and clinical laboratories can now detect gene expression patterns with prognostic value (64). We developed an integrative pipeline to construct a GSCS using the expression profiles of GSCs marker genes. We validated this signature in six independent cohorts and confirmed that the best model is a combination of VSOLassoBag and RSF. The algorithm combinations eliminated low-value features, optimized the model, and enhanced its generalization ability (65). The GSCS performed well in predicting outcomes across all six cohorts, as shown by ROC and C-index analyses, indicating its potential clinical utility. To maximize the clinical utility of the GSCS, future research should focus on integrating this biomarker into routine diagnostic workflows. Investigating its application in personalized treatment plans could enhance therapeutic decision-making, particularly for patients with varying glioma subtypes. Additionally, exploring the GSCS in conjunction with existing prognostic factors may lead to more refined risk stratification models. Longitudinal studies assessing the signature’s predictive power in response to specific therapies will be crucial. Furthermore, expanding the signature’s validation across diverse populations and treatment settings will strengthen its relevance and applicability in clinical practice, ultimately improving patient outcomes.

Based on the RSF algorithm, TUBA1C was identified as the most significant gene in GSCS. TUBA1C is an isoform of α-tubulin that has been shown to play a critical role in the cell cycle and immune microenvironment of lung adenocarcinoma (LUAD). Elevated TUBA1C expression correlates with poor outcomes and with tumor-infiltrating immune cells (TIICs) in LUAD (66). Additionally, TUBA1C is upregulated in hepatocellular carcinoma (HCC) and pancreatic ductal adenocarcinoma (PDAC), where it predicts poor prognosis and enhances cell proliferation and migration (67, 68). Furthermore, a prior study indicated that TUBA1C was statistically associated with the expression of RP11-480I12.5 in breast cancer (BRCA) and demonstrated prognostic significance (69). TUBA1C has also been shown to promote aerobic glycolysis and cell growth via upregulation of YAP expression, thereby contributing to BRCA development. Our findings further revealed that TUBA1C knockdown significantly inhibited the malignant biological behaviors of glioma cells, demonstrating that TUBA1C is a promising target for the treatment of glioma.

This study has advanced our understanding of GSCs and their clinical relevance; however, we acknowledge several limitations. First, the cohorts exhibited had heterogeneity due to different in sequencing or microarray platforms. We harmonized the data using standard normal transformation, which was only partially effective. Second, we relied on retrospective samples, necessitating future validation in a prospective, large cohort. Third, we should conduct more in-depth and detailed molecular biology studies in both *in vivo* and *in vitro* experiments to uncover the molecular mechanisms of tumor recurrence and identify new therapeutic targets.

## Conclusion

This study provides a comprehensive characterization of GSCs through integrated analysis of single-cell RNA sequencing, spatial transcriptomics, and machine learning approaches. We identified a distinct GSC population with high proliferative potential and developed a novel 26-gene GSCS that exhibits robust prognostic value across multiple cohorts. The signature demonstrated pan-cancer prognostic ability and an association with critical tumorigenic pathways. We validated the functional significance of TUBA1C, a key component of our signature, through *in vitro* and *in vivo* experiments. Silencing TUBA1C significantly inhibited glioma cell proliferation, migration, and invasion, as well as tumor growth in xenograft models. This study enhances our understanding of glioma biology and provides a clinically relevant prognostic tool and potential therapeutic targets.

## Data Availability

The datasets presented in this study can be found in online repositories. The names of the repository/repositories and accession number(s) can be found in the article/**Supplementary Material**. The Raw data is available at: https://www.jianguoyun.com/p/DWH4qUEQ45m7Cxih8NwFIAA.

## Supplementary material

Supplementary Figure 1
**(A)** Determination of the number of trees by minimizing error. **(B)** Variable importance of the top 26 genes determined using the random survival forest (RSF) algorithm.

Supplementary Figure 2 Relative TUBA1C mRNA expression levels in xenograft tumors from si-NC and si-LN299 groups. qPCR analysis revealed that TUBA1C expression was significantly reduced in the si-LN299 group compared to the si-NC group. ***p < 0.001.
