# Supplementary figures and images for: From single-cell to spatial transcriptomics: decoding the glioma stem cell niche and its clinical implications
Source: Front Immunol. 2024 Sep 17;15:1475235. doi: 10.3389/fimmu.2024.1475235 (PMC11443156; doi:10.3389/fimmu.2024.1475235)

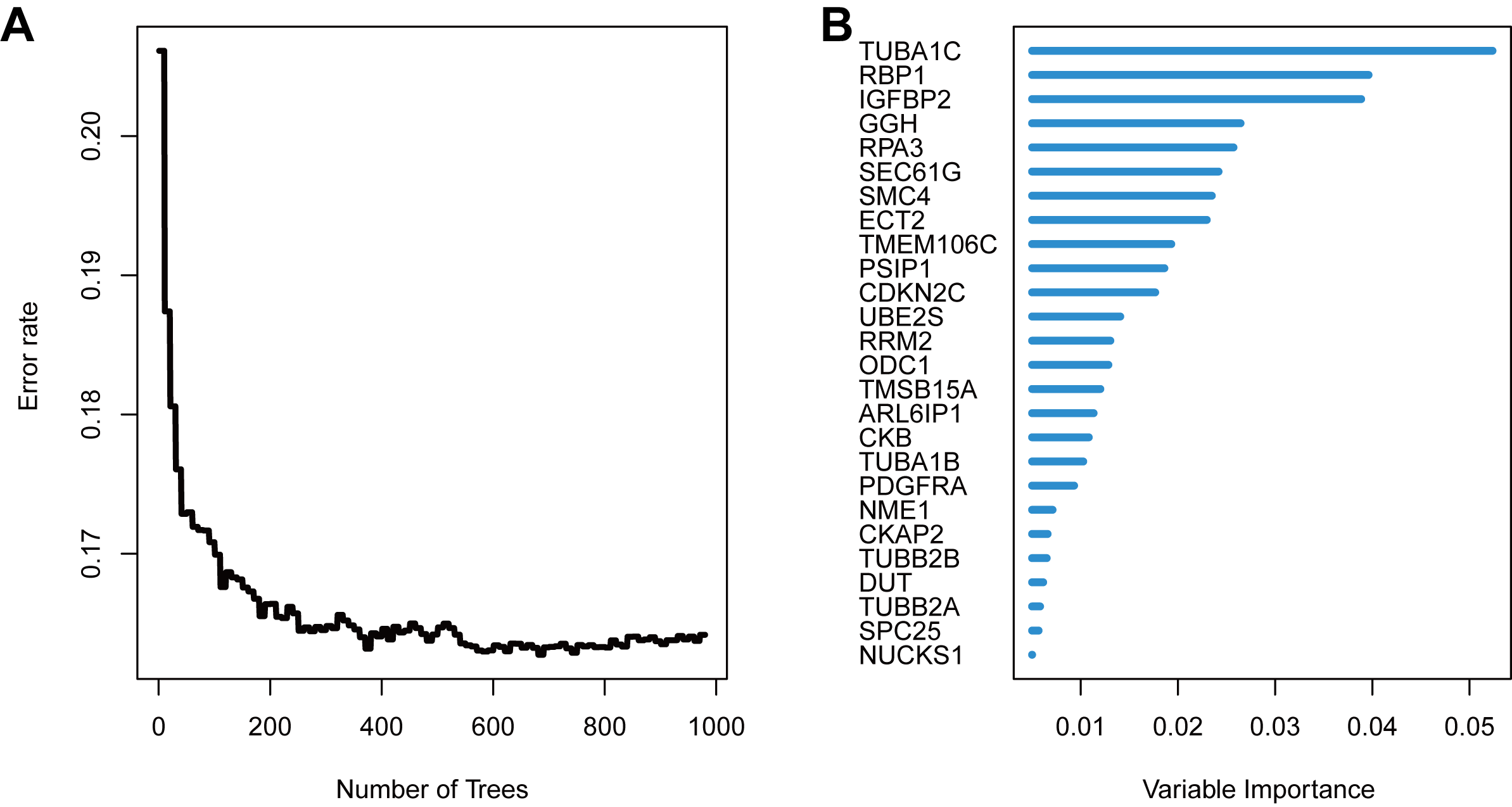

Supplement: Supplementary Figure 1 — (A) Determination of the number of trees by minimizing error. (B) Variable importance of the top 26 genes determined using the random survival forest (RSF) algorithm. [file Image1.tif]

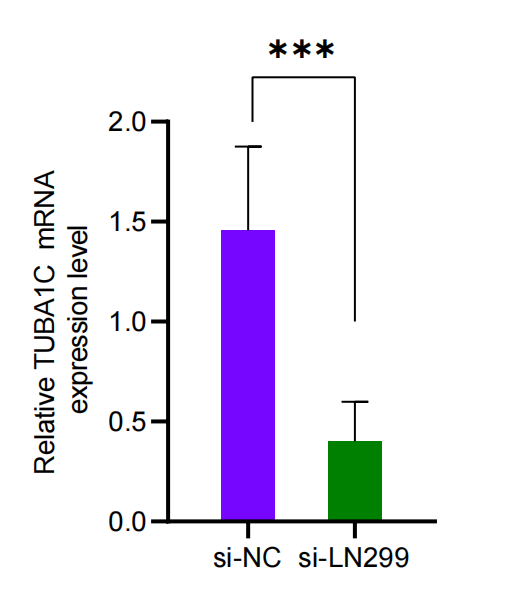

Supplement: Supplementary Figure 2 — Relative TUBA1C mRNA expression levels in xenograft tumors from si-NC and si-LN299 groups. qPCR analysis revealed that TUBA1C expression was significantly reduced in the si-LN299 group compared to the si-NC group. ***p < 0.001. [file Image2.tif]
