# Supplementary material for: From single-cell to spatial transcriptomics: decoding the glioma stem cell niche and its clinical implications
Source: Front Immunol. 2024 Sep 17;15:1475235. doi: 10.3389/fimmu.2024.1475235 (PMC11443156; doi:10.3389/fimmu.2024.1475235)
Supplement: Supplementary file 3 [file Table1.docx]

| **Oligonucleotides** | **Nucleotide sequence (5'-3')** |
| --- | --- |
| **siRNA** |  |
| Si-TUBA1C-1 | CCCAACCTACACTAACCTTAA |
| Si-TUBA1C-2 | CCCACAGTCATTGATGAAGTT |
| **Primer** |  |
| TUBA1C | GGGATGAGTGCTTTGTGTGC (forward) |
|  | CCGTGTTCCAGGCAGTAGAG (reverse) |

**Table S1. Oligonucleotides used in research**
